# The safety and feasibility of extracorporeal high-intensity focused ultrasound (HIFU) for the treatment of liver and kidney tumours in a Western population

**DOI:** 10.1038/sj.bjc.6602803

**Published:** 2005-09-27

**Authors:** R O Illing, J E Kennedy, F Wu, G R ter Haar, A S Protheroe, P J Friend, F V Gleeson, D W Cranston, R R Phillips, M R Middleton

**Affiliations:** 1 HIFU Unit, Churchill Hospital, Oxford OX3 7LJ, UK; 2 Clinical Center for Tumor Therapy, Chongqing University of Medical Sciences, People's Republic of China; 3 Institute of Cancer Research, Sutton, UK; 4 Cancer Research UK, Medical Oncology Unit, University of Oxford, Oxford, UK

**Keywords:** ablation, clinical, HIFU, kidney, liver

## Abstract

High-intensity focused ultrasound (HIFU) provides a potential noninvasive alternative to conventional therapies. We report our preliminary experience from clinical trials designed to evaluate the safety and feasibility of a novel, extracorporeal HIFU device for the treatment of liver and kidney tumours in a Western population. The extracorporeal, ultrasound-guided Model-JC Tumor Therapy System (HAIFU™ Technology Company, China) has been used to treat 30 patients according to four trial protocols. Patients with hepatic or renal tumours underwent a single therapeutic HIFU session under general anaesthesia. Magnetic resonance imaging 12 days after treatment provided assessment of response. The patients were subdivided into those followed up with further imaging alone or those undergoing surgical resection of their tumours, which enabled both radiological and histological assessment. HIFU exposure resulted in discrete zones of ablation in 25 of 27 evaluable patients (93%). Ablation of liver tumours was achieved more consistently than for kidney tumours (100 *vs* 67%, assessed radiologically). The adverse event profile was favourable when compared to more invasive techniques. HIFU treatment of liver and kidney tumours in a Western population is both safe and feasible. These findings have significant implications for future noninvasive image-guided tumour ablation.

Over the last decade, minimally invasive therapies have developed to provide a role in the ablation of solid tumour deposits in the liver and kidney. Radiofrequency waves (Gervais *et al*, 2003), cryotherapy (Sotsky and Ravikumar, 2002), lasers (Vogl *et al*, 2004), microwave energy (Shibata *et al*, 2002), ethanol injection (Livraghi *et al*, 1995) and high-intensity focused ultrasound (HIFU) (Kennedy, 2005) have all been used to ablate tumours; however, HIFU remains the only modality to be completely extracorporeal.

Ultrasound may pass harmlessly through human tissue, yet when focused at high intensities, sufficient energy may be deposited to produce a well-demarcated volume of coagulation necrosis, independent of soft tissue type (Hill *et al*, 1994). This has been known since the 1940s (Lynn *et al*, 1942); however, it is only recently that advances in imaging have allowed accurate placement of the acoustic focus and thus its clinical exploitation (Kennedy *et al*, 2003).

The most widely used clinical extracorporeal device is the Model-JC HIFU System (Chongqing HAIFU™ Company). The device has been used in China and the Far East since 1997 (Wu *et al*, 2004). Recent data suggest a survival advantage when it is used in combination with trans-arterial chemoembolisation (TACE) in patients with unresectable hepatocellular carcinoma (Wu *et al*, 2005a). There is also data to show that HIFU has a role to play in the relief of intractable pain relating to pancreatic cancer (Wu *et al*, 2005b). We report results from early phase II studies investigating the safety and feasibility of extracorporeal HIFU for the treatment of liver and kidney tumours in a Western population using this system.

## PATIENTS AND METHODS

**Figure 1:**
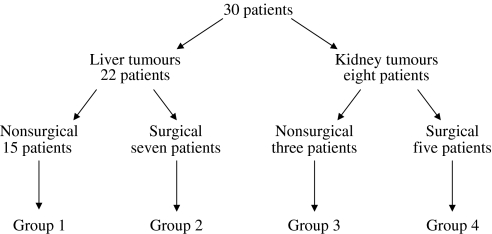
Patient disposition.

All trials were approved by the Oxfordshire Clinical Research Ethics Committee and conformed to GCP guidelines. Patients included both surgical and nonsurgical candidates and each patient had one or more solid tumour deposits in either the liver or kidney (see Figure 1). All patients were over 18 years old and gave written informed consent to take part in the study. Previous surgery, chemo- and biological therapy were permitted provided that they had recovered from any related side effects. No patients had received radiotherapy to the target region in the preceding 12 months. Patients were required to have normal bone marrow function (haemoglobin ⩾10 g dl^−1^, absolute neutrophil count ⩾1500 mm^−3^, platelet count ⩾100 000 mm^−3^), renal function (urea and creatinine <2.5 times upper limit of laboratory normal (ULN) range) and adequate hepatic reserve (prothrombin time ⩽1.5 times ULN, activated partial thromboplastin time ⩽1.5 times ULN, total bilirubin <1.5 times ULN, AST ⩽3 times ULN, alkaline phosphatase <2 times ULN, unless arising from the bone). All patients required an American Society of Anaesthesiologists grade of ⩽2, and a World Health Organization performance status of ⩽1.

General exclusion criteria for all trials included: women who were pregnant or nursing, clinical evidence of brain metastases, subjects with tumours lying <5 mm from vital structures, concurrent antiarrhythmic, anticoagulant or immunosuppressive medication, permanent implanted pacemakers and those who had previously documented severe intra-abdominal adhesions.

The Model-JC HIFU System (Chongqing HAIFU™ Company) was used in all cases and has been described previously (Kennedy *et al*, 2004). Briefly, the device has a 12 cm diameter, single element, piezo-ceramic transducer fronted by acoustic lenses of varying focal lengths, driven at 0.8 or 1.6 MHz. An AU3 US imaging device (Esaote, Genoa, Italy) is mounted coaxially with the high-energy transducer allowing treatment to be guided in real time.

Patients received gadolinium contrast-enhanced (CE) magnetic resonance imaging (MRI), baseline blood tests and symptom review prior to HIFU therapy. Each patient received a single HIFU treatment session under general anaesthesia; a double lumen endotracheal tube allowed single lung ventilation, thus minimising organ movement during respiration. Treatment consisted of a combination of single and multiple overlapping ultrasonic pulses directed to the target tumour. According to the trial protocols, a single tumour, or part of a single tumour, was selected for ablation. Grey scale changes visualised on B-mode diagnostic ultrasound during treatment allowed assessment of tissue response. At the time of HIFU treatment, the estimated dimensions of the ablated region were recorded in the anteroposterior (AP), transverse and cranial–caudal dimensions. This provided the reference value by which both radiological and histological observations were assessed.

Radiological follow-up for all patients was at 12 days (or within 3 days of day 12) with a further CE-MRI. The MRI sequences used in the evaluation and quantification of ablation varied according to the target organ. Liver tumours were assessed using the reformatted FAME (Fast Acquisition, Multiple Excitation) sequences 1 min postinjection of intravenous gadolinium contrast agent. Kidney tumours were assessed using subtraction films, where the FAME precontrast series was taken from the 1 min postcontrast series. Repeat blood analyses and symptom reviews took place on days 1, 2 and 12. Surgical resection of the tumours in groups 2 and 4 took place within 14 days of HIFU treatment. Patients in groups 1 and 3 had a symptom review on day 30 after treatment.

Primary end points of the study were adverse events and variations in clinical laboratory data during the first 28 days following treatment (or until surgical resection). Device-related adverse events were graded according to the Common Toxicity Criteria (Version 2, Final 1/30/98) where applicable.

Secondary end points were radiological and histological assessment of response. Radiological response was evaluated in terms of the presence and accuracy of ablation within the target tumour. ‘Accuracy’ was assessed from the 12-day post-HIFU MRI images. Where ablation was seen, the Radiologist commented on whether the zone fell within the target tumour (‘good’) or outside the target tumour (‘poor’). The size of any zone of coagulation necrosis was measured – these were given both as short-axis diameter (the minimum requirements according to the Working Group on Image-Guided Tumour Ablation special report; Goldberg *et al*, 2003) and also area estimates derived from the AP and transverse measurements. ‘Radiological response’ was derived from a comparison between the maximal area of ablation seen on MRI and the maximal intended area of ablation recorded at the time of treatment. It should be noted that the intended area of ablation did not necessarily equate to the patient's total tumour burden.

Pathological evaluation of the treated lesion followed surgery in groups 2 and 4. At the time of surgery, the specimen was marked, and oriented in a way that enabled histological slices to correspond as far as possible with MRI slices. Sections (5 mm) were cut and photographed to allow semiquantitative assessment of histological response. Microscopic confirmation of the effect of HIFU was obtained by taking representative histological slides from areas, which included interfaces between tissues of different macroscopic appearance. These interfaces included those between any areas of necrotic and nonnecrotic tumour, between viable tumour and normal liver and between necrotic and viable liver. The assessment compared the maximal area of ablation seen on macroscopic vital staining with the maximal estimated area of ablation recorded at the time of treatment.

## RESULTS

Between November 2002 and August 2004, 30 patients (23 male, seven female) were recruited with mean age 65 years (range 40–84 years). Of these, 22 patients had liver metastases (18 colorectal, one breast, one lung and two adenocarcinoma of unknown primary). All kidney tumours were renal cell carcinoma. All the patients were treated with HIFU and were evaluable for adverse events.

Table 1 summarises the treatment parameters. During the research period, treatment times were limited to approximately 2 h. This constraint limited the extent to which a given tumour that could be treated in any single HIFU session.

A total of 15 patients were treated with HIFU in group 1. One treatment session was abandoned when the water reservoir housing the treatment head leaked. One patient died 10 days after HIFU of causes unrelated to the HIFU treatment. Although no imaging was performed post-HIFU, the patient underwent post-mortem examination and the results of this evaluable in terms of histological response. As a result, 13 of the 15 patients have been evaluated for MRI response to treatment. All 15 patients were evaluable for device-related adverse events.

In the liver surgery group (group 2), the seven enrolled patients all completed the HIFU treatment session and had both the pre- and post-HIFU MRI. All were evaluable for device-related adverse events. One patient did not proceed to surgery when further liver metastases were discovered on pre-HIFU I.

In group 3, two of the three patients had a complete HIFU treatment session and were evaluable in terms of radiological response. One session was terminated prematurely as bowel had become interposed in the treatment field. All three were evaluable in terms of adverse events.

All five patients in group 4 completed the HIFU treatment session; however, on retrospective independent radiological analysis, one patient was thought to have a renal scar rather than a renal tumour. This patient did not proceed to surgical resection and was excluded from the final radiological analysis.

Device-related adverse recorded for the trial patients are summarised in Table 2. Although the majority of patients experienced some discomfort, this was generally transitory. Those requiring additional opiates were only administered them in the immediate post-HIFU period. All patients felt well enough to be discharged from hospital the following day as per the protocol.

**Figure 2:**
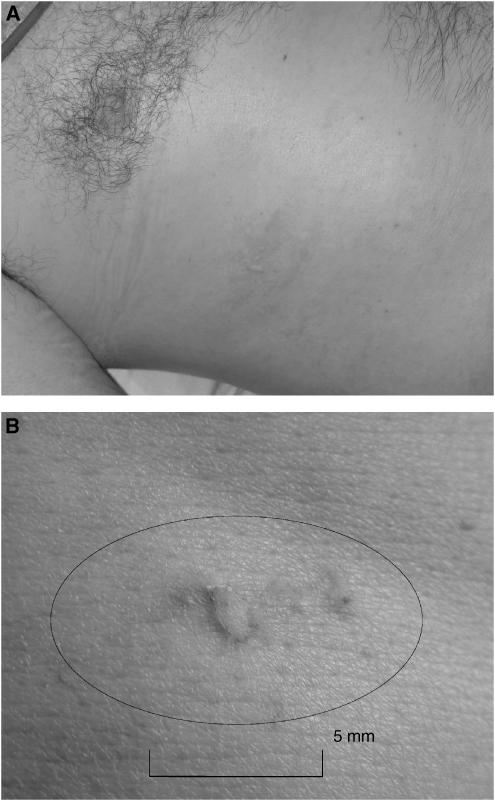
(**A**) Right chest wall. Grade 1 skin toxicity following intercostal HIFU treatment to a liver metsastasis. (**B**) Close-up of lesion showing scale.

The most common form of skin toxicity was the formation of a blister or track at the treatment site, <1 cm diameter. These were typically 1 × 1 mm across, as shown in Figure 2A and B. Skin toxicity was treated with cool-packs and aloe gel: grade 1 toxicity generally resolved by day 12 without further action. Grade 2 toxicity (partial thickness blister or burn >1 cm diameter) also required no further treatment, but took longer to resolve. No grade 3 skin toxicity was seen. Oedema at the treatment site was transitory, generally resolving prior to discharge with no further treatment. Four patients developed a mild fever during the first 24 h after treatment, never above 40°C. All fevers settled with antipyretics and had resolved prior to discharge. One patient experienced increased skin sensitivity at the treatment site, and a further patient had some permanent subcutaneous thickening and pigmentation at the treatment site.

There was a transient and clinically insignificant drop in haemoglobin immediately after HIFU (mean decrease of 1.0 g dl^−1^ (range 0.9–3.2 g dl^−1^)). As there has been no blood loss associated with the treatments, this is likely to be a dilutional artefact following anaesthesia. A transient rise was also seen in white blood counts immediately after treatment (mean increase of 1.71 × 10^−9^ l^−1^ (range −8.10 to 9.94 l^−1^)), and CRP values on day 2 were also raised (median increase of 9.8 mg l^−1^ (range −52 to 70 mg l^−1^)). These generally small and transient rises from baseline imply a mild, nonspecific inflammatory process. No changes were seen in biochemical markers of renal function in either the liver or kidney tumour trials. There was no change in liver function in those patients treated in groups 2, 3 or 4. Those patients who had deterioration in liver function were all part of group 1, and had liver disease that was inoperable. Clinical disease progression was seen in seven of these patients; biochemical progression was also noted in seven patients. There were transient rises in bilirubin of two patients, both of which settled at day 12. One patient had a slow rise in bilirubin (not associated with an immediate post-treatment spike), most likely related to cancer progression. AST did rise transiently after treatment in 15 of the 22 patients (mean rise of 22.6 IU l^−1^ on day 2 post-HIFU) as might be expected from a small volume of cellular destruction. ALP rose gradually in six patients, and LDH in five cases, but in these instances, there was no transient rise immediately post-HIFU.

**Figure 3:**
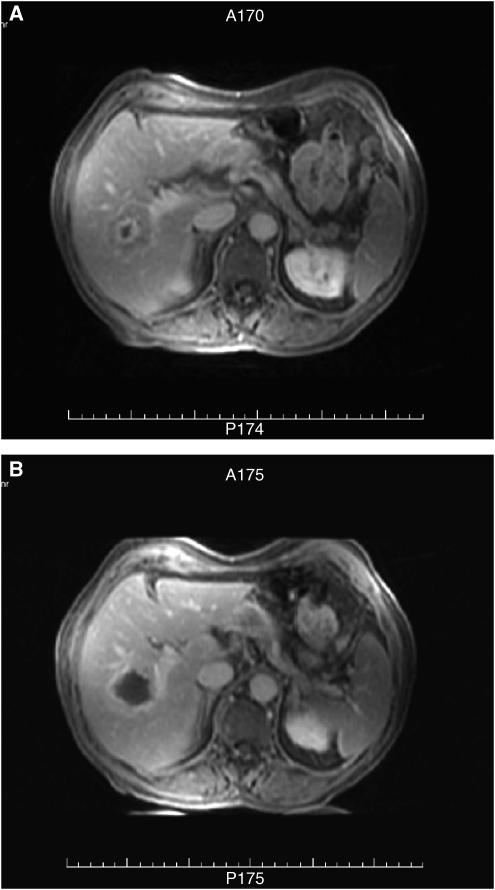
Axial FAME series MRI images, 1 min post IV gadolinium contrast. (**A**) Before HIFU with a right hepatic metastasis within segment VIII showing central necrosis, and (**B**) 12 days after HIFU, a larger zone consistent with coagulation necrosis within the metastasis.

**Figure 4:**
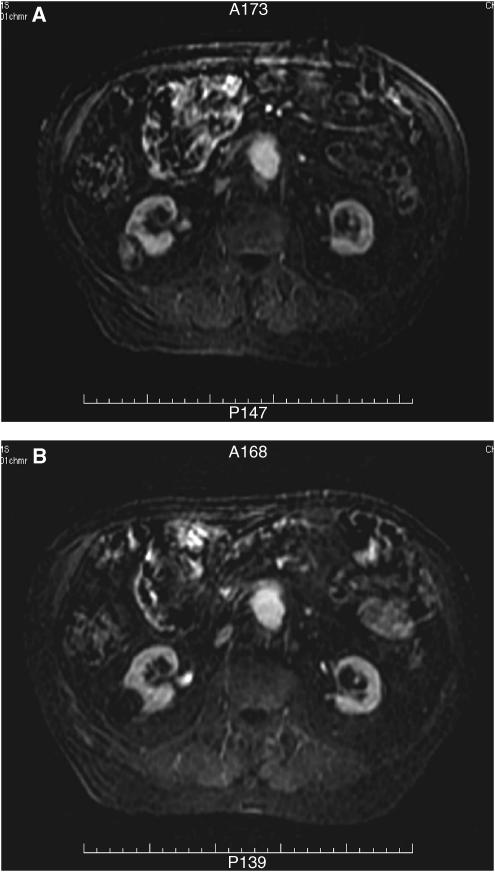
Subtraction MRI films, taking the FAME precontrast series from the 1 min postcontrast images. Patient with a right primary renal tumour (**A**) before HIFU showing contrast uptake within the target tumour, and (**B**) 12 days after HIFU, showing no contrast uptake within the target tumour, consistent with ablation.

Of the 30 patients treated, 27 were evaluable in terms of response to treatment. Evidence of ablation was seen (radiologically or histologically) in 25 patients (93%). Of the 26 patients who had radiological evaluation, 24 had clear zones of ablation on post-HIFU MRI. Accuracy was assessed as ‘good’ in 21. Two patients had zones of ablation lying 2 mm in front of the target tumour, and one patient had a zone of ablation lying 2 mm beyond the target. The data for overall radiological and histological evaluation are given in Table 3. Examples of radiological evidence of ablation are shown in Figures 3 and 4, for liver and kidney tumours, respectively.

## DISCUSSION

High-intensity focused ultrasound is in its infancy in the West. The work carried out in China suggests that this modality has great potential in the treatment of solid tumour deposits. We have shown that extracorporeal HIFU is both a safe and feasible option for cancer patients, with a favourable side-effect profile.

All adverse events were local to the treatment site and self-limiting. The only clinically relevant symptom encountered has been discomfort, which although reported in 80% of cases has generally been ‘mild’ in severity. Skin toxicity was seen in eight cases (27%). In seven of these, toxicity consisted of pinhead blisters or tracks that were not clinically relevant and resolved spontaneously. Grade 2 skin toxicity was seen only in one case, which also resolved spontaneously. Subcutaneous oedema occurred in eight of those treated; however, none of these occurrences were clinically relevant, and settled spontaneously. The working party on image-guided tumour ablation identifies ‘postablation syndrome’ consisting of a self-limiting sign complex of low-grade fever and general malaise. In all, 13% of those treated experienced a low-grade fever, consistent with postablation syndrome. This occurred within the first 12 h and had settled within 24 h. There are other potential complications of HIFU, including damage to adjacent viscera such as bowel or gallbladder, and secondary infection of the resulting necrotic volume, but these have not been observed in our patients.

Changes in laboratory values demonstrate that the physiological consequences of HIFU upon hepatic and renal function are minimal, even when those organs are the target. Creatinine levels have been unaffected in the renal tumour group, an important consideration if HIFU is to be administered to those with poor renal reserve. Likewise, hepatic function remained stable after HIFU, with the exception of those patients with gross metastatic infiltration of the liver, where a decline in liver function might be expected. The transient elevation in white cell count and CRP seen immediately after HIFU are not clinically significant, and likely reflect a systemic inflammatory response to the tumour ablation.

We have demonstrated that HIFU exposure results in the creation of discrete zones of ablation in 25 of 27 targeted solid tumour deposits. Accuracy, as assessed by MRI, showed that the lesions were placed directly on the target tumour in 88% of cases, and in the remainder, the ablated region lay within 5 mm of the target. It is important to put this into clinical context, as during any cancer surgery or ablation, tumours are excised or ablated along with a surrounding margin of normal tissue, usually no less than 1 cm. On the assumption that similar principles would apply to HIFU treatment, all observed zones of ablation occurred with these margins.

The radiological analysis of HIFU- (Table 3) treated liver tumours shows that the median area of ablation seen on MRI is 45% smaller than that predicted at the time of treatment. Although this disparity would seem to be large, the histological zones of ablation correlate more closely with those expected. The median area of histological ablation is 102% of the median intended area.

Liver tumours were ablated more reliably than kidney tumours. This may be due to the greater depth of renal tumours and the presence of a perinephric fat layer, both of which attenuate the ultrasonic beam. For the purpose of analysis, only those renal tumours that showed evidence of ablation either radiologically or histologically are included in the analysis of expected *vs* measured response. Again, the difference between median area of ablation seen on MRI and intended is greater than that seen between histological ablation and intended (25 *vs* 6%, respectively).

There are potential limitations to the clinical application of HIFU, and to the planning and the actual delivery of treatment. HIFU cannot be directed through air-filled viscera such as the lung or bowel and other obstructions such as bone can absorb or reflect an ultrasound beam. For this reason, tumours in the dome of the liver are not likely to be suitable targets for HIFU, unless further invasive procedures are performed, such as injection of saline into the pleural cavity to produce an acoustic window (F Wu, personal communication). Ablation of tumours lying in close proximity to bowel or gall bladder run the risk of visceral perforation should patient movement occur during treatment. Even in the absence of such complicating factors, if a target tumour is situated at a depth greater than 10 cm from the skin, the attenuation of the normal tissues in the beam-path reduces the likelihood of successful ablation with current devices. Additional factors such as obstruction of the incident ultrasound energy by the ribs or reflection by tissue interfaces can also lead to undertreatment. Potential advantages and limitations are summarised in Table 4. Another concern is that HIFU may promote the spread of metastases. Small animal studies have shown that HIFU does not increase the risk of metastatic spread (Oosterhof *et al*, 1997), and there is evidence that ablative therapies may upregulate the response of host immune system to subsequent tumour challenge (Yang *et al*, 1992; den Brok *et al*, 2004).

Treatment times are longer than is desirable. A treatment session lasting for 2 h for a superficial 2–3 cm tumour may be acceptable when compared to the alternative of surgical resection, but compares less favourably with other minimally invasive techniques such as radiofrequency ablation. For the treatment of large tumours, where there is no minimally invasive alternative option, the longer treatment times may be justified on the grounds of a lower morbidity and mortality than conventional surgery. Despite this, it is likely that treatment times will reduce with development of the technology, experience and in combination with methods to reduce tumour perfusion, such as trans-arterial embolisation (Wu *et al*, 2005a).

In our series, HIFU was performed under general anaesthesia to ensure patient comfort and immobility. This is generally regarded as a limitation, but general anaesthesia does provide a means to control respiratory excursion in organs such as the liver and kidney. Movement of these organs during HIFU exposure could compromise treatment efficacy and preventing this motion overcomes what would otherwise be a further limitation of HIFU.

Surgery for the treatment of liver and kidney tumours carries a significant morbidity and mortality, and may be associated with long in-patient stays and recovery periods. Extracorporeal HIFU offers the same potential for disease control as other minimally invasive treatments, and has the benefit of being noninvasive. In contrast to radiotherapy, treatments can in principle be repeated, as there is no ceiling to the number of ultrasound exposures that normal tissues can tolerate if not at the focus of the HIFU beam.

Other groups are also generating results in different areas; an MRI-guided extracorporeal HIFU device (Exablate, Insightec Inc., Haifa, Israel) has been granted FDA approval for the treatment of uterine fibroids (Stewart *et al*, 2003), and trans-rectal HIFU devices are increasingly being used to treat localised prostate cancer in Europe (Blana *et al*, 2004).

High-intensity focused ultrasound holds promise; however, randomised control studies are required to evaluate the true potential of this novel modality. There are many areas in which HIFU may provide benefit: both in the curative setting when compared to open or minimally invasive procedures and in palliation, where quality of life may be improved. The studies presented here document the safety and efficacy of this novel treatment in a preliminary group of patients and should provide a basis for the next phase of trials to begin.

## Figures and Tables

**Table 1:** Patient treatment parameters

	**Mean**	**Range**
Anaesthetic time (min)	209	150–271
Patient positioning (min)	17	8–14
Time to locate tumour and plan treatment (min)	46	8–133
Treatment duration (min)	123	30–189
Total exposure (min)	20	0.2–43.5

**Table 2:** Adverse events possibly or probably related to HIFU treatment^a^

	**CTC grade**			
**Event**	**0**	**1**	**2**	**3**
Discomfort at treatment site	6	16	7	1
Skin toxicity at treatment site	22	7	1	0
Oedema at treatment site	22	3	3	2
Fever	26	3	1	0
Other	27	3	0	0

HIFU=high-intensity focused ultrasound; CTC=common toxicity criteria.

a Number of patients who experienced each adverse event by grade.

**Table 3:** Results of the HIFU trials^a^

		**Intended area of ablation (cm^2^)**		**Measured area of ablation (cm^2^)**	
	**Ablation seen in**	**Median**	**Range**	**Median**	**Range**
*Liver*					
Radiologically assessed	20/20 (100%)	4.8	0.8–9.0	3.3	0.1–42.9
Histologically assessed	6/6 (100%)	5.5	3.8–7.5	5.6	0.2–16.0
					
*Kidney*					
Radiologically assessed	4/6 (67%)	4.0	1.5–6.0	5.3	0.8–9.7
Histologically assessed	1/4 (25%)	4.5	—	4.8	—

HIFU=high-intensity focused ultrasound.

a Comparison of the expected area of ablation at the time of treatment with that measured on follow-up imaging or histology.

**Table 4:** Benefits and limitations of HIFU

**Benefits**	**Limitations**
Noninvasive	Requires general anaesthetic
Safe – morbidity much less than surgery	Long time taken to ablate given volume
Real-time imaging allows evaluation of area during treatment	Position of tumours affects ability to treat
Large scope for treatment of different tumour types	Local pain, oedema and skin toxicity
No risk of increased metastasis	
Potential host immune upregulation	
Potentially curative	
Repeatable	

HIFU=high-intensity focused ultrasound.
